# Context-dependent duality of senescent cells in liver fibrosis

**DOI:** 10.1016/j.livres.2026.09.001

**Published:** 2026-09-04

**Authors:** Yuqi Gu, Linjing Zhan, Yaping Di, Haipeng Wu, Tiejun Zhao, Fei Zhang, Qirou Wu

**Affiliations:** a School of Medicine, Hangzhou City University, Hangzhou, Zhejiang, China; b College of Pharmaceutical Science, Zhejiang University, Hangzhou, Zhejiang, China; c Institute of Intelligent Medicine, ZJU Hangzhou Global Scientific and Technological Innovation Center, Hangzhou, Zhejiang, China

**Keywords:** Cellular senescence, Liver fibrosis, Hepatic stellate cell (HSC), Senescence-associated secretory phenotype (SASP), Antifibrotic therapy

## Abstract

Liver fibrosis, marked by excessive deposition of extracellular matrix (ECM), is a critical precursor to cirrhosis, liver failure, and hepatocellular carcinoma. Accumulating evidence highlights that cellular senescence, characterized by stable cell-cycle arrest and senescence-associated secretory phenotypes (SASPs), is a key regulator of the fibrotic process. This review systematically dissects the context-dependent roles of senescent cells in liver fibrosis, with a focus on hepatocytes, hepatic stellate cells (HSCs), liver sinusoidal endothelial cells (LSECs), Kupffer cells, and natural killer (NK) cells. Distinct populations of senescent cells emerge sequentially during different stages of fibrosis, exhibiting divergent kinetics and functional effects. The SASP, a dynamic cocktail composed of cytokines, chemokines, and proteases, remodels the intrahepatic microenvironment through immune cell recruitment, regulation of cell function, and modulation of ECM turnover. These effects vary depending on their molecular composition and disease context. Furthermore, we discuss emerging senescence-targeted therapeutic strategies to restore liver homeostasis. In-depth understanding of the cell-type and stage-specific roles of senescence in fibrosis provides a molecular foundation for translating senescence-targeted strategies into clinical precision antifibrotic therapies.

## Key Highlights

• Cellular senescence exerts context-dependent dual roles in liver fibrosis, acting as either a driver or a restraint depending on the specific cell type and context involved.
• The SASP dynamically remodels the hepatic microenvironment by modulating immune responses and inducing paracrine senescence.
• Precision therapeutic strategies should selectively eliminate pathogenic senescent cells while sparing or inducing beneficial senescence.

## Graphical abstract

**Figure:**
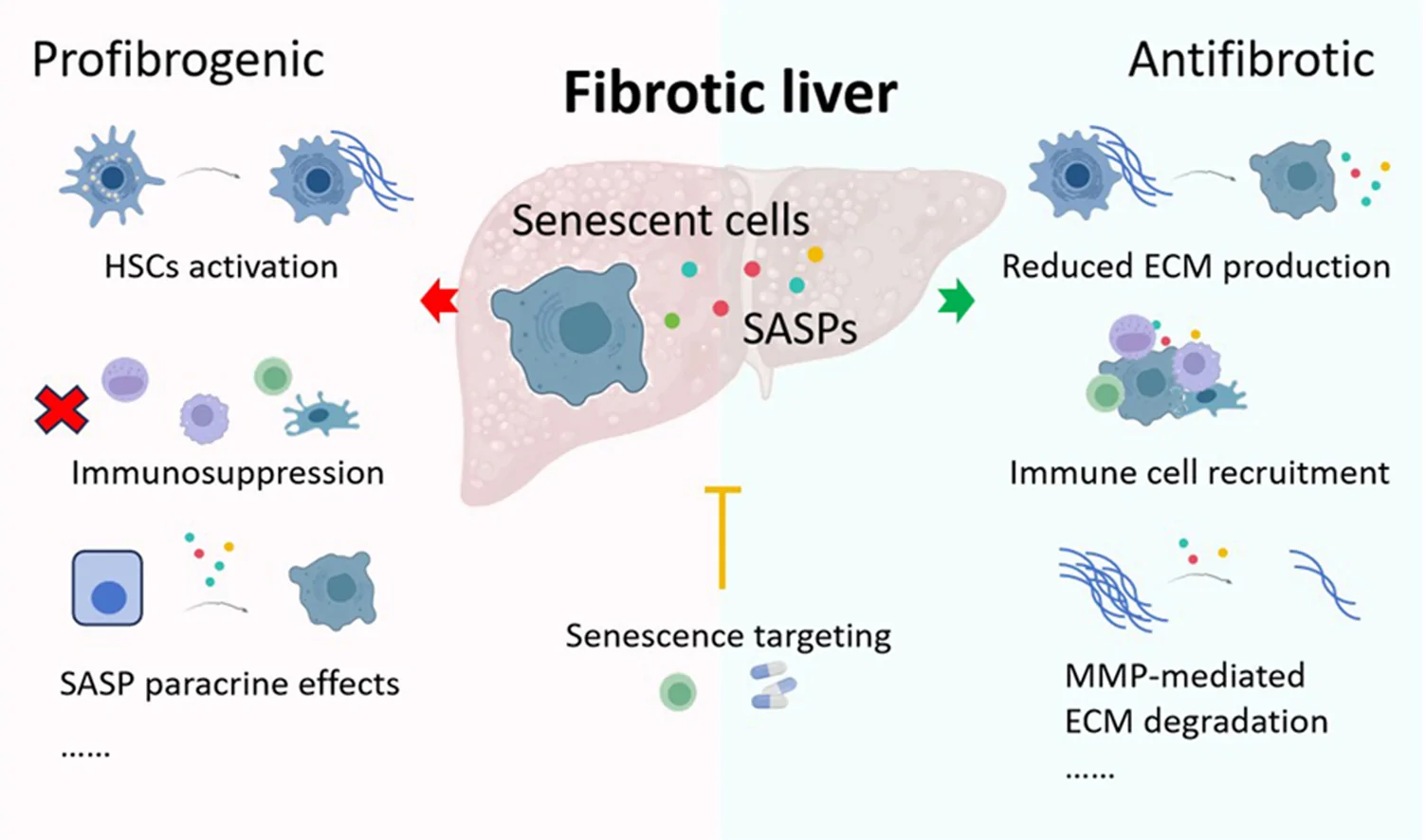

## 1 Introduction

Liver fibrosis is characterized by the excessive accumulation of extracellular matrix (ECM), mainly cross-linked type I and type III collagens, leading to the formation of fibrous scars.^1^ It is typically initiated by sustained hepatic insults, ranging from viral infections, alcohol abuse, and drug- or toxin-induced injury, to endogenous drivers such as metabolic dysfunction, cholestatic disorders, acute or chronic inflammation, and the pro-fibrotic milieu of aging.^2^ When damage occurs, related cytokines and growth factors are released, which activate the primary effector hepatic stellate cells (HSCs). Activated HSCs secrete various factors to remodel liver architecture and drive the progression of fibrosis. The development of liver fibrosis arises from a dynamic and intricate interplay among a diverse array of cell populations, including HSCs, hepatocytes, endothelial cells and immune cells.^2^^,^^3^ Persistent liver fibrosis can progress to liver cirrhosis, ultimately leading to irreversible liver failure. Besides, liver fibrosis is strongly linked to liver cancers.^4^ Notably, current treatments for liver fibrosis are often insufficient to achieve complete fibrosis reversal.^5^

Beyond the paradigm of HSC activation, cellular senescence has recently emerged as a critical player in liver fibrosis.^6^, ^7^, ^8^ Cellular senescence is defined by stable cell-cycle arrest, which can be triggered by a wide spectrum of exogenous and endogenous stressors, including telomere erosion, oxidative damage, mitochondrial dysfunction and oncogenic signaling. Senescent cells are always accompanied by a spectrum of characteristic features like senescence-associated secretory phenotype (SASP), which actively remodels the tissue microenvironment.^9^ During the progression of acute and chronic liver diseases, senescent cells emerge and progressively accumulate in the hepatic microenvironment. Liver injury from viral hepatitis, alcohol abuse, or metabolic dysfunction-associated steatohepatitis (MASH) triggers senescence of multiple cell types, including HSCs, hepatocytes, Kupffer cells, and cholangiocytes.^10^ Currently, research into cellular senescence in liver fibrosis is transitioning from broad descriptions of senescent functions toward a more nuanced understanding of the specific cell populations involved.^11^^,^^12^ However, a comprehensive spatiotemporal map remains largely elusive. Critical questions persist regarding exactly which hepatic lineages undergo senescence, their distinct functional roles, and how these dynamics vary across different fibrotic models and stages.^10^^,^^13^ Central to this uncertainty is the poorly characterized heterogeneity of senescent cells across diverse liver lineages. For instance, in murine liver fibrosis models, senescence of activated stellate cells limits liver fibrosis.^14^ Conversely, clearance of senescent macrophages significantly mitigates hepatocellular damage.^11^ This paradox underscores that cellular senescence in liver fibrosis is not a monolithic driver of pathology nor a uniformly beneficial process. Consequently, a major unmet challenge in the field lies in determining how to achieve the selective elimination of pathogenic senescent clusters while sparing those with reparative functions.^15^^,^^16^ Resolving these knowledge gaps represents the critical frontier for eventually translating senotherapies into precise antifibrotic strategies.

In this review, we aim to dissect the stage-specific emergence of senescent hepatocytes, HSCs, endothelial cells and immune cells during fibrosis. We discuss how these senescent populations differentially drive or restrain fibrosis through SASP-mediated immune modulation, ECM remodeling and paracrine senescence propagation. Human data and murine studies underscore that the effect of senescence depends on cell type, disease setting and disease stage. Induction or clearance strategies for cellular senescence need to be adapted in a highly context-specific manner. This mechanistic framework anchors the rational translation of senolytic strategies into patient-tailored antifibrotic therapies.

## 2 Cellular heterogeneity and functional diversity of senescence in liver fibrosis

Cellular senescence in liver pathology shows dynamic heterogeneity, with distinct senescent cell populations appearing at different disease stages. In acute hepatic injury, hepatocytes predominantly undergo stress-induced senescence, functioning as sentinels through damage-associated molecular pattern (DAMP) release to initiate tissue repair.^17^ Chronic liver damage induces a more complex senescence landscape, where Kupffer cell senescence initiates a pro-inflammatory cascade that subsequently triggers senescence in HSCs, hepatocytes, and cholangiocytes, a process critically involved in fibrogenesis.^18^ Notably, HSCs represent a context-dependent double-edged identity. Senescent HSCs down-regulate alpha-smooth muscle actin (α-SMA) and type I and type III collagen, leading to reduced ECM accumulation.^14^^,^^19^ However, if compromised immune surveillance allows senescent HSCs to evade clearance and accumulate, they develop a potent SASP. The SASPs can reshape the liver microenvironment by promoting immune cell infiltration, sustaining inflammation, and inducing secondary senescence in neighboring non-senescent cells.^12^^,^^13^ During cirrhosis evolution, the senescence signature shifts to include endothelial cells and macrophages, contributing to microvascular dysfunction and inflammatory exacerbation.^20^ Intriguingly, in hepatocellular carcinoma, while malignant hepatocytes escape senescence, the persistent senescent stroma may paradoxically support tumor progression through SASP-mediated microenvironment remodeling.^21^ These intricate regulatory networks render the role of cellular senescence in hepatic fibrogenesis exquisitely cell-type-dependent (Fig. 1).

**Fig. 1:**
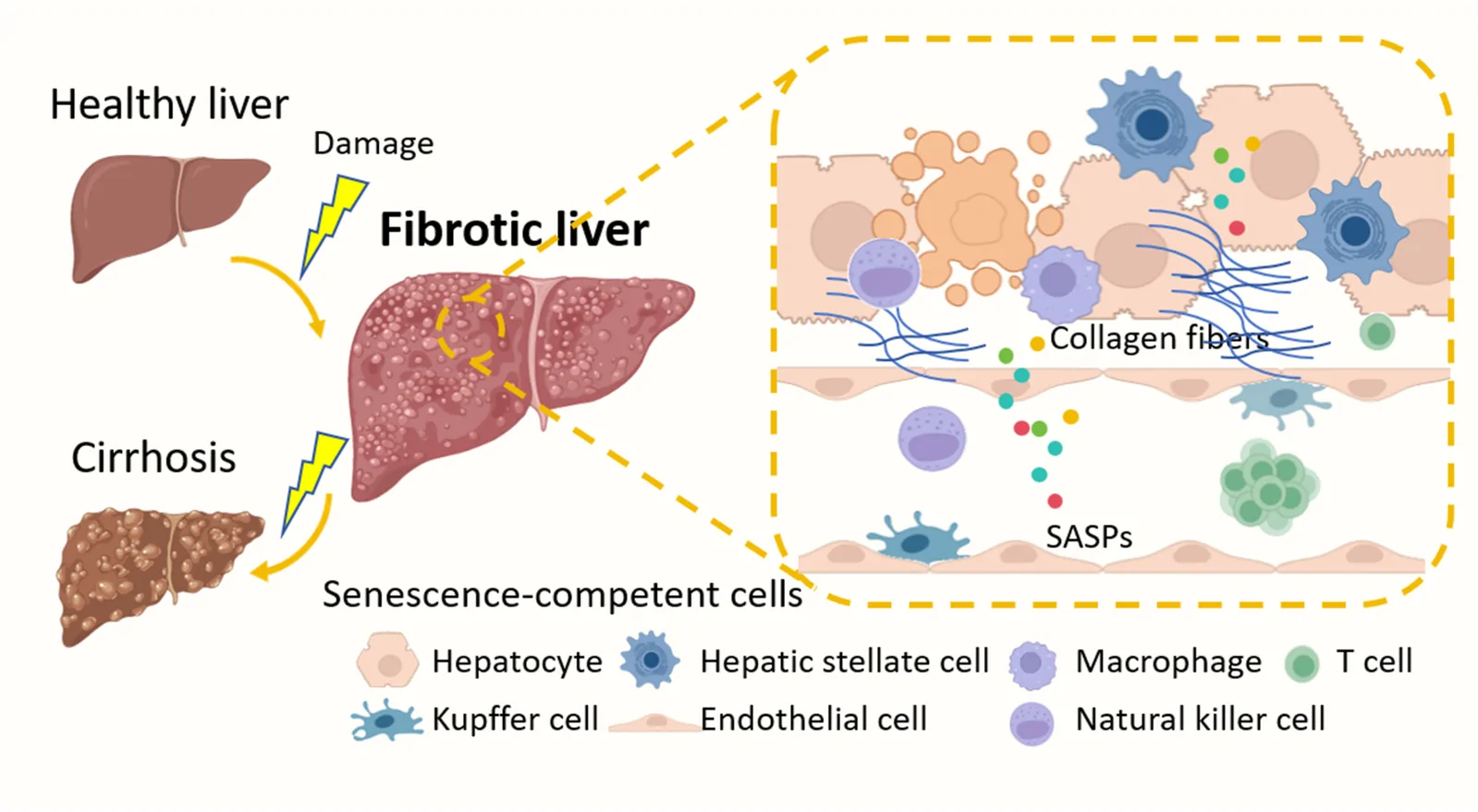
**Senescent cells in fibrotic liver.** Persistent hepatic injury drives the progression from fibrosis to cirrhosis. This damage not only activates hepatic stellate cells (HSCs) to deposit collagen and remodel the extracellular matrix (ECM), but also induces phenotypic switching and cellular senescence. In the fibrotic liver, multiple cell types such as hepatocytes, HSCs, sinusoidal endothelial cells, and immune populations undergo senescence. In addition, the secretion of senescence-associated secretory phenotypes (SASPs) alters the hepatic microenvironment.

However, this dynamic landscape is not solely dictated by the stage of liver injury but is profoundly modulated by systemic host factors. In the context of aging, immunosenescence impairs the clearance of senescent cells by NK cells and macrophages, leading to their pathological accumulation even after the initial insult has resolved.^22^ Age-related mitochondrial dysfunction disrupts metabolic homeostasis and triggers sterile inflammatory responses via DAMPs to drive fibrosis progression, highlighting that enhancing mitochondrial quality control offers a promising strategy to ameliorate liver fibrosis.^23^^,^^24^ Thus, a deeper understanding of senescence mechanisms is essential to develop effective interventions that combine the targeted elimination of specific senescent cell subsets with the restoration of a conducive microenvironmental milieu.

### 2.1 Hepatocyte senescence

Hepatocytes, as the primary parenchymal cells of the liver and key players in various metabolic processes, are highly susceptible to diverse chronic injuries due to their vigorous metabolic activity.^25^ Under different pathological conditions, hepatocytes may incur issues like DNA damage, oxidative stress, metabolic overload and iron accumulation, and thus enter a state of senescence.^26^ Additionally, replicative stress induced by chronic injury or ageing leads to shortening of telomeres, rendering ageing a significant contributor to hepatocyte senescence.^27^ In patients with metabolic dysfunction-associated steatotic liver disease (MASLD) and MASH, and their corresponding animal models, senescent hepatocytes are commonly found in fibrotic zones and periportal regions, indicating their spatial relevance in the initiation of fibrogenic signaling.^28^

In the early stages of liver fibrosis, senescence in hepatocytes is a potent mechanism driving liver fibrogenesis. Senescent hepatocytes exhibited elevated expression of cell cycle inhibitors such as p21, increased DNA damage signaling, and telomere shortening. These changes collectively induced irreversible cell cycle arrest and reduced responsiveness to key mitogenic signals such as hepatocyte growth factor (HGF) and epidermal growth factor (EGF).^29^ As a result, the regenerative potential of hepatocytes was markedly diminished. This loss of regenerative ability impaired liver tissue repair and facilitated the progression of fibrosis, ultimately exacerbating the chronic liver disease state.^29^ The function of hepatocyte senescence is highly context-dependent.^27^ SASPs comprise pro-fibrotic and pro-inflammatory cytokines such as transforming growth factor (TGF)-β, interleukin-6 (IL-6), C-X-C motif chemokine ligand (CXCL)-10 and C–C motif chemokine ligand 2 (CCL2). Some SASP factors recruit immune cells to the site of injury for immune clearance, thereby playing a beneficial role in wound healing as a transient response.^30^ For instance, senescent hepatocytes promote NK cell migration dependent on the CXCL-10/CXCR3 axis.^31^ Oncogene-induced senescence also occurs in normal hepatocytes *in vivo*. Pre-malignant senescent hepatocytes secrete chemokines and cytokines and are recognized and cleared by CD4^+^ T-cell-mediated adaptive immune responses.^32^ However, some SASP factors act in a paracrine manner to activate HSCs, facilitating their *trans*-differentiation into collagen-producing myofibroblasts.^16^^,^^33^ In addition, senescent hepatocytes can also promote immune responses by promoting macrophage migration, which may drive hepatocarcinogenesis.^34^^,^^35^

### 2.2 Senescence in hepatic stellate cells

HSCs are liver-specific pericytes residing in the space of Disse, serving as the central regulators in the complex pathogenesis of liver fibrosis. Quiescent HSCs are vitamin A reservoirs but transdifferentiate into myofibroblast-like cells upon liver injury. These activated HSCs secrete massive amounts of type I collagen and fibronectin, driving fibrotic deposition and hepatic architectural disruption.^36^ Subsequently, activated HSCs undergo p53-dependent senescence. Rather than simply halting their ECM production, these senescent HSCs switch to secreting SASPs like matrix metalloproteinases (MMPs) and pro-inflammatory cytokines, which actively remodel the microenvironment and can enhance fibrosis regression.^14^ Senescent HSCs have been detected in different types of liver fibrosis, including animal models induced by alcohol, MASLD, viral hepatitis, carbon tetrachloride (CCl_4_) intoxication, and bile duct problems, suggesting a conserved underlying mechanism linking HSC senescence to the fibrotic process (Fig. 2).^37^

**Fig. 2:**
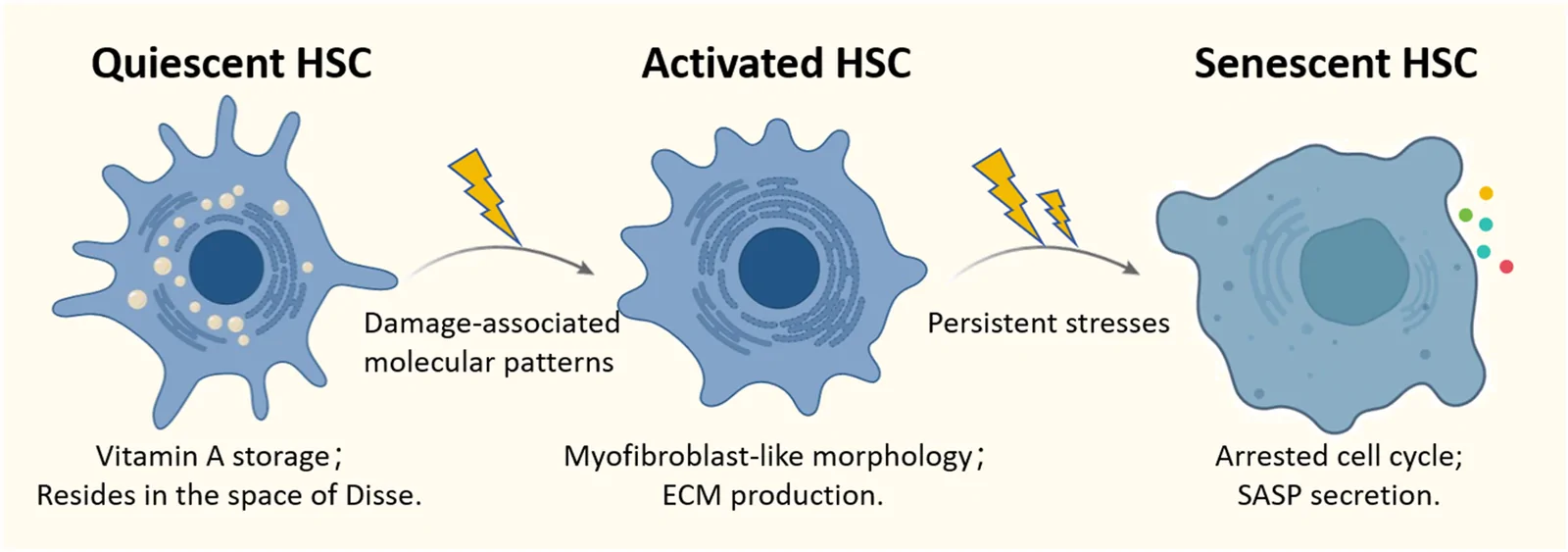
**Cellular senescence of HSC in liver fibrosis.** Schematic depiction of the sequential phenotypic states of HSCs during fibrotic liver injury. Quiescent HSCs reside in the space of Disse, and store vitamin A. Activated HSCs secrete abundant type I collagen and fibronectin, driving ECM accumulation. Persistent stresses and damage-associated molecular patterns (DAMPs) trigger senescence of HSCs, which arrest the cell cycle and the secrete senescence-associated secretory phenotypes (SASPs).

Early studies have shown that senescent HSCs markedly reduce ECM production, positioning the induction of HSC senescence as a promising strategy to limit the progression of liver fibrosis.^14^ Indeed, specific attenuation of HSC senescence in fibrotic livers, without eliminating the cells themselves, paradoxically exacerbates fibrosis severity.^38^ This finding underscores that the senescence program itself acts as an essential physiological brake on myofibroblast activity. In addition, some SASPs like MMP-9 and MMP-13 help degrading fibrotic collagen deposits.^39^ However, the narrative is not unilaterally positive; HSC senescence is intrinsically double-edged. While SASPs secreted by senescent cells can recruit multiple immune cell subsets, the heterogeneity and persistence of the SASP can drive pathology. Senescent cells also produce non-classical MHC molecule HLA-E, which inhibits NK and CD8^+^ T cell-mediated immune responses.^40^ When immunosurveillance is compromised, persistent senescent cells can establish a chronic, low-grade, pro-fibrotic inflammatory milieu accompanied by immune dysregulation, thereby further amplifying senescent cell accumulation.^22^ Furthermore, the paracrine effects of SASP components like IL-6, tumor necrosis factor-alpha (TNF-α) induce secondary senescence in hepatocytes and cholangiocytes via p53/p21 and p16/Rb pathways, impairing hepatic function and amplifying inflammation.^41^^,^^42^ While SASP-driven paracrine senescence exacerbates microvascular dysfunction and parenchymal damage, its cell-autonomous induction of HSC senescence creates a counterregulatory mechanism.^43^ This duality underscores a critical therapeutic challenge to maintain the delicate balance between beneficial and detrimental senescence. Therefore, selectively targeting pro-fibrotic senescent cells remains a formidable challenge.

Cellular senescence of HSCs can be regulated by multiple factors and signaling pathways. Oxidative stress, a key inducer, triggers DNA damage in HSCs through excessive reactive oxygen species (ROS), activating the p38 MAPK pathway to upregulate senescence markers like p21 and p16, as seen in CCl_4_-induced liver injury models where ROS elevation correlates with increased HSC senescence.^44^ Additionally, the cyclic GMP-AMP synthase (cGAS)-stimulator of interferon genes (STING) pathway has emerged as a novel regulator: cytosolic DNA from damaged or infected cells activates this pathway, triggering type I interferon production and HSC senescence, as demonstrated *in vitro* by increased SA-β-gal expression following cGAS-STING agonist treatment.^45^ These mechanisms highlight the complex interplay between stress signals and signaling cascades in governing HSC senescence during liver fibrosis. Another crucial factor is the TGF-β signaling pathway. While TGF-β is a major driver of HSC activation and fibrosis in the early stage, persistent TGF-β stimulation can also trigger HSCs senescence. In bile duct ligation (BDL)-induced liver fibrosis model, blocking TGF-β signaling attenuated HSCs senescence, suggesting its essential role in promoting senescence under fibrotic conditions.^46^ The regulation of HSC senescence could greatly promote beneficial senescence or eliminate the therapeutic potential of senescent cells in the treatment of liver fibrosis. HSC senescence serves as a key self-limiting mechanism in liver fibrosis, shifting activated HSCs to a senescent state that reduces ECM deposition and recruits immune cells, with beneficial effects from SASP factors outweighing paracrine drawbacks.

### 2.3 Senescence in sinusoidal endothelial cells

LSECs are the major EC type in the liver, and are in a fully differentiated state, with fenestrae, lack of an organized basement membrane, and active endocytosis. They function to regulate material exchange between hepatocytes and blood, phagocytize pathogens and apoptotic cells in the blood.^47^ Dysregulation of LSECs promotes pathological processes within the liver including inflammation, microvascular thrombosis, and fibrosis.^48^ Under normal circumstances, LSECs can inhibit the activation of HSCs and promote hepatocyte proliferation by enhancing the release of nitric oxide (NO) and increasing the expression of hepatocyte growth factor and members of the Wnt gene family.^49^ When the liver is damaged, LSECs undergo senescence and capillarization, and the secretion of alpha-2 glycoprotein 1 (LRG1) increases. This can upregulate the expression of α-SMA and type I collagen through the TGF-β signaling, thereby promoting liver fibrosis. At the same time, it can activate the IL-6/JAK/STAT3 pathway and secrete a large number of inflammatory mediators, further aggravating liver fibrosis.^50^ In etoposide-induced senescent LSECs, the expression levels of the anti-aging protein sirtuin 1 (SIRT1), endothelial nitric oxide synthase (eNOS), and phosphorylated eNOS are decreased. This leads to endothelial dysfunction, impairs intrahepatic microcirculation, increases intrahepatic vascular resistance, promotes the formation of portal hypertension, and thereby drives the progression of liver fibrosis.^51^ The principal pathogenic consequence of senescent LSECs is the establishment of persistent portal hypertension, a hemodynamic stress that is sustained by their pseudocapillarization-dependent increase.

### 2.4 Kupffer cell senescence

Kupffer cells, the liver's resident macrophages, defend against pathogens and tune inflammation, playing a critical role in maintaining hepatic homeostasis. They can be recruited by senescent cells, and then undergo polarization and senescence.^52^^,^^53^ M2-polarised Kupffer cells have been shown to drive IL-6-dependent senescence in hepatocytes via activation of the STAT3-p53-p21 axis, which can serve as a protective mechanism during early stages of alcoholic liver disease.^54^ M2-polarised macrophages can also activate HSCs to transform into myofibroblasts by secreting cytokines such as TGF-β and platelet-derived growth factor (PDGF), promoting the progression of fibrosis.^55^ Senescent Kupffer cells weaken their phagocytic activity to clear damaged cells, indirectly promoting fibrosis.^2^ After specifically depleting p16^+^ macrophages which are senescent by genetic tools, the expression of intrahepatic fibrosis markers and fibrotic areas are significantly reduced, indicating that depletion of senescent macrophages can effectively alleviate liver fibrosis.^11^

Besides, age-related alterations in Kupffer cells further compound their contribution to fibrosis. In schistosomiasis-induced hepatic injury, Kupffer cells exhibited reduced phagocytic activity and antigen presentation capacity, correlating with sustained granulomatous inflammation and diminished senescence clearance.^56^ Moreover, CD11b-positive macrophages infiltrate the injured liver and express elevated levels of Fas ligand (FasL) and TNF-α, contributing to hepatocyte apoptosis, resulting in severe tissue damage.^57^ In addition, Nrf2 signaling in Kupffer cells and LSECs has reduced oxidative stress and dampened inflammatory responses during fibrosis, indicating a multicellular regulatory effect.^58^^,^^59^

### 2.5 Senescence in natural killer cells

NK cells are crucial regulators of hepatic fibrogenesis, primarily through the targeted elimination of damaged, activated or senescent hepatic cells. Senescent HSCs express elevated levels of NKG2D ligands, including Rae1 and Mult1, rendering them susceptible to NK-mediated cytotoxicity via perforin and granzyme B granule exocytosis.^60^^,^^61^ This senescence surveillance mechanism is impaired during ageing, as demonstrated by reduced mitochondrial function and cytolytic capacity of liver-resident NK cells. Importantly, rosuvastatin treatment reverses NK cell senescence by restoring mitochondrial fitness, reestablishing their antifibrotic potential in aged murine models.^62^ In MASH, senescent hepatocytes were found to secrete C-X-C motif chemokine ligand 10 (CXCL-10). This chemokine recruits CXCR3^+^ NK cells, thereby enhancing local immune clearance of damaged cells and contributing to fibrosis attenuation.^63^ However, chronic liver injury leads to NK cell exhaustion, marked by reduced IFN-γ production and upregulation of inhibitory receptors such as NKG2A. In HIV/HCV coinfection, advanced fibrosis correlates with NK dysfunction that is not rescued by CD4^+^ T cell helper, suggesting an irreversible senescence-like state.^64^

### 2.6 Senescence in other hepatic cell populations

In addition to the aforementioned cell types, other cellular populations within the liver, including T cells, dendritic cells (DCs), and endothelial cells, also undergo senescence-related changes that contribute to the progression of liver fibrosis. T cells undergoing senescence display reduced cytokine secretion and elevated PD-1 expression, leading to compromised effector functions and failed immunosurveillance of senescent cells.^64^ The senescent microenvironment also affects DCs, especially the CD103^+^ subset within the liver. Under chronic injury conditions such as MASLD, these cells exhibit impaired maturation and antigen presentation capacity. This dysfunction weakens their ability to activate cytotoxic lymphocytes, limiting the immune system's ability to recognize and eliminate senescent cells.^65^ And depletion of senescent endothelial cells aggravated liver injury.^11^ Furthermore, recent findings have identified a novel mechanism by which senescent immune cells evade immune clearance. Specifically, they upregulate ganglioside-based immune checkpoints. These checkpoints mediate immune evasion to prevent recognition by NK and T cells. This immune evasion strategy contributes to the accumulation of senescent cells in the liver, particularly in the elderly or those with chronic liver disease.^66^ The regulation of liver fibrosis by senescent cells is tightly linked to the intrinsic roles of the cell types. Furthermore, SASPs, a key mechanism by which senescent cells modulate the microenvironment, exert highly divergent regulatory effects on liver fibrosis progression via their constituent factors.

## 3 Systemic microenvironmental remodeling by SASP in liver fibrosis

The SASPs play a central role in coordinating cellular senescence, immune responses, and the development of liver fibrosis.^67^ SASP factors profoundly reshape the intrahepatic immune microenvironment by influencing both immune and parenchymal cell functions. Proinflammatory cytokines such as TNF-α and IL-6 can recruit monocytes and promote their polarization into proinflammatory M1 macrophages, which exacerbate the intrahepatic inflammatory response by releasing more pro-fibrotic mediators, such as TGF-β and ROS.^68^^,^^69^ In contrast, IL-10 in SASP can induce the polarization of M2 macrophages, indirectly enhancing ECM degradation via macrophage-derived MMPs, thus exerting antifibrotic effects.^52^ Chemokines CCL2 and IL-8 further contribute by recruiting CCR2^+^ monocytes, T lymphocytes, and neutrophils into fibrotic regions; the former sustains adaptive immune activation, whereas neutrophil infiltration leads to tissue injury through protease and ROS release.^42^^,^^70^ Proteolytic enzymes such as MMP-9 and MMP-13 can degrade fibrotic collagen, but excessive or unbalanced activation disrupts ECM homeostasis and aggravates tissue injury.^71^ Together, these events create a self-sustaining inflammatory microenvironment.

One of the key functions of SASP in liver fibrosis is the recruitment of immune cells. For instance, in murine models of CCl_4_-induced liver injury, TNF-α–dependent signals were found to facilitate hepatic DCs maturation and monocyte-derived macrophage infiltration, contributing to fibrotic progression.^72^ They also attract inflammatory immune cells, particularly macrophages, into the fibrotic niche, a specialized microenvironment characterized by activated HSCs, excessive ECM deposition, and sustained inflammation, thereby accelerating fibrosis progression. One of the key SASP components, CCL2, plays a pivotal role by recruiting CCR2^+^ monocytes and facilitating their differentiation into pro-inflammatory macrophages.^73^^,^^74^

Beyond immune cell recruitment, SASP factors such as TGF-β-modulate immune responses through paracrine signaling. TGF-β not only induces senescence in adjacent cells, but also promotes fibrogenesis by activating both M2-polarised macrophages and HSCs.^53^ Notably, TGF-β is stored in the ECM in a latent form and requires mechanical or enzymatic activation, often triggered by ECM stiffening during fibrosis.^75^ Active ECM remodeling also in fibrosis releases DAMPs, such as fibronectin fragments, low-molecular-weight hyaluronic acid, and biglycan, which serve as ligands for pattern recognition receptors like TLR2, TLR4 on immune cells. These ECM-derived DAMPs actively modulate immune responses by enhancing cytokine release, promoting further immune cell activation, and perpetuating chronic inflammation.^76^^,^^77^

After establishing the pro-inflammatory effects of SASP, it is equally crucial to comprehensively evaluate its immunosuppressive and anti-inflammatory roles, which may contribute to fibrosis resolution.^78^ Components such as TGF-β and IL-10 not only induce the differentiation and immunosuppressive capacity of regulatory T cells (Tregs) but also influence the activation, polarization, and senescence of various immune cells, including macrophages and dendritic cells.^52^^,^^79^ It contributes to immune homeostasis, tissue repair, and the senescence of various immune cells, forming a complex immunoregulatory network.^80^ Moreover, SASP produced by senescent HSCs can promote M1 macrophage polarization and enhance immune surveillance. A murine model of liver fibrosis demonstrated that senescent HSCs restricted fibrosis progression through immune surveillance.^14^ Immune-mediated clearance of senescent cells, driven by a properly balanced SASP composition, is critical for maintaining liver homeostasis and suppressing tumor development.^81^ However, prolonged exposure to SASP during chronic senescence can impair this regulation by disrupting Treg functions and shifting the Th17/Treg balance, a hallmark of chronic inflammation and fibrogenesis.^30^^,^^82^ For instance, in CCl_4_-induced murine liver fibrosis models, an elevated Th17/Treg ratio has been positively correlated with fibrosis severity.^83^ These findings underscore that SASP shapes the fibrotic immune microenvironment through multiple mechanisms, including promoting Treg differentiation, modulating macrophage polarization, enhancing dendritic cell antigen presentation, and altering Th17/Treg balance.

Overall, SASP exerts a dual role in immune regulation, with outcomes determined by its molecular composition and signaling duration. Targeting SASP to modulate immune cell recruitment and restore immune homeostasis offers a promising therapeutic avenue for chronic liver diseases, potentially reducing fibrosis and boosting anti-tumor immunity.^84^ Further translational studies are warranted to evaluate the safety and efficacy of SASP-targeted interventions in patients with MASH, cirrhosis, or early-stage hepatocellular carcinoma.^85^^,^^86^

## 4 Targeting cellular senescence as a therapeutic strategy for liver fibrosis

The therapeutic targeting of cellular senescence offers a promising avenue for reversing liver fibrosis. Indeed, senolytic approaches such as combination dasatinib plus quercetin (D + Q) and pan-urokinase-type plasminogen activator receptor (uPAR)-directed chimeric antigen receptor (CAR) T cells can eliminate senescent cells to reduce fibrosis and restore liver function.^87^, ^88^, ^89^ However, the simplistic idea of eliminating all senescent cells carries risks across different stages of fibrogenesis. Given that senescent cells may also play constructive roles to promote tissue remodeling,^15^ and the cell-type-specific roles of senescent cells in fibrogenesis, effective strategies will likely need to encompass both pan-senolytic agents and interventions targeted to specific hepatic cell populations.

Targeting hepatocyte senescence has emerged as a potential antifibrotic strategy. Insulin-like growth factor-1 (IGF-1) administration has been shown to restore mitochondrial membrane potential, suppress p53 activation, and enhance hepatocyte proliferation, thereby alleviating senescence-associated damage in ageing and fibrotic livers.^90^^,^^91^ Notably, IGF-1 exhibits indirect antifibrotic effects by modulating HSC activation via PI3K/AKT signaling, thereby suppressing collagen synthesis and maintaining ECM homeostasis in both animal and co-culture models.^92^^,^^93^ Despite these benefits, IGF-1 signaling is a potent driver of liver cancer stem cell (CSC) systemic administration in cirrhotic patients could theoretically accelerate hepatocellular carcinoma (HCC), outweighing its antifibrotic benefits.^94^ This highlights the critical need for context-specific delivery or transient modulation to avoid oncogenic transformation. Similarly, Nrf2 activation by Alda-1 has been shown to enhance mitochondrial function, ROS accumulation, and upregulate antioxidant enzymes, thereby suppressing hepatocyte senescence and attenuating fibrosis progression in aging and injured livers.^95^ Iron accumulation in senescent hepatic cells has also been shown to amplify SASP-related inflammation, while iron chelation with deferiprone shifts the SASP toward an anti-inflammatory profile, attenuating fibrosis progression. Iron chelators such as deferiprone have effectively reduced hepatocyte cell senescence and fibrosis without affecting HSCs.^26^ Under metabolic stress, a distinct subset of senescent hepatocyte gene signature (SHGS)-positive hepatocytes emerges from a p21-positive progenitor state. These senescent cells undergo a functional collapse, losing key hepatic activities while secreting pro-fibrotic factors such as growth differentiation factor 15 (GDF 15), whose circulating levels correlate tightly with both SHGS ^+^ burden and disease progression. Crucially, pharmacological intervention with a senolytic agent DpC, a second-generation thiosemicarbazone and Dp44mT analog, selectively eliminates this SHGS^+^ population, together with fibrosis in a mouse model of MASLD.^6^ Consequently, reversing hepatocyte cell senescence through metabolic and mitochondrial reprogramming or neutralizing SASP signaling via cytokine blockade provide mechanistic support for next-generation antifibrotic therapies, provided that safety profiles are rigorously optimized.

Emerging senescence-targeted interventions offer promising alternatives to traditional therapies that solely target HSC activation. Antioxidants such as N-acetylcysteine (NAC) can reduce ROS levels, inhibit p38 mitogen-mediated senescence and promoting HSC apoptosis, thereby reducing fibrosis in CCl_4_-induced liver injury model.^96^^,^^97^ N-acetylcysteine 2 (Nrf2) activators can not only inhibit hepatocyte senescence but also block p38 MAPK-driven HSC senescence.^95^^,^^98^ Beyond modulating the senescence microenvironment, delivery systems are being developed to actively induce senescence in activated HSCs. A prime example is the albumin-mediated transcytosis delivery of manganese, a cGAS-STING agonist. This approach specifically triggers senescence in activated HSCs, thereby inhibiting the progression of liver fibrosis.^7^ However, inducing senescence therapeutically poses a paradox: if not strictly controlled, these induced senescent cells may persist and secrete a pro-fibrotic SASP, necessitating a follow-up senolytic clearance. Immune checkpoint blockade of the PD-1/PD-L1 axis has been explored, and in a MASH model, blocking PD-L1 enhances the clearance of senescent HSCs by cytotoxic T lymphocytes and accelerates fibrosis regression.^99^ However, uncontrolled T-cell activation and cytokine release can trigger immune-related adverse events (irAEs) affecting multiple organs, among which immune-related liver injury is particularly common.^100^ Additionally, genetic reprogramming to reverse HSC senescence restores endothelial function and reduces scar formation in murine fibrosis models.^11^ Additionally, Hippo-YAP signaling governs HSC fibrogenic activity, selective YAP deletion triggers HSC senescence and renders them more susceptible to senolytics.^101^ Targeting hepatic senescence via oxidative stress modulation or immune checkpoint blockade shows promise for treating liver fibrosis by enhancing this antifibrotic processes.

Therapeutic strategies targeting endothelial cells are primarily aimed at managing portal hypertension. A long-time course of simvastatin in aged cirrhotic rats enhanced the hepatic sinusoidal environment, resulting in a marked reduction in portal hypertension.^102^ The selective ROCK2 inhibitor TDI01 restores LSEC fenestration and suppresses pro-fibrotic angiocrine signaling in early clinical trials.^103^ Administering the peroxisome proliferator-activated receptor (PPAR) agonist lanifibranor targeting LSECs and perivascular HSCs, the fenestration can be improved, and at the same time reducing the level of liver fibrosis in advanced chronic liver disease.^104^ Moreover, miR-325–3p helps restore LSEC fenestrations by modulating their cytoskeleton, effectively reversing capillarization and significantly reducing fibrosis in mouse models of liver fibrosis.^105^ For aged immune cells, agents including prostaglandin E receptor 3 (EP3) blockers, alcohol dehydrogenase 3 (ADH3) inhibitors, and natural killer group 2 member D (NKG2D) agonists have been shown to restore partial immune-cell functionality and exert anti-fibrotic effects in the liver.^106^

In summary, cellular senescence is no longer a single-cell problem but a multicellular, context-dependent driver of liver fibrosis. As senolytics, SASP-normalizers, and immune-restorative agents enter early-phase trials, the field must pivot from merely demonstrating efficacy to rigorously addressing the risk-benefit balance (Table 1).^6^^,^^7^^,^^26^^,^^87^^,^^88^^,^^90^^,^^95^^,^^96^^,^^98^^,^^99^^,^^102^^,^^104^^,^^107^ While the emerging therapies discussed herein offer significant promise, a critical appraisal of their potential limitations, off-target toxicities, and formidable barriers to clinical translation is essential to guide the development of safe and effective antifibrotic regimens.

**Table 1:** Senescence-targeted therapeutic strategies for liver fibrosis.

Drug/Intervention	Target/Mechanism of action	Target cells	Model	Effect on liver fibrosis	Reference/NCT No.
Dasatinib plus quercetin	Induces apoptosis in senescent cells	Senescent cells	Metabolic dysfunction-associated steatotic liver disease (MASLD)/metabolic dysfunction-associated steatohepatitis (NASH) models	Reduces fibrosis	NCT05506488
Urokinase-type plasminogen activator receptor (uPAR)-targeting chimeric antigen receptor (CAR) T	Clears senescent cells	Senescent cells	Carbon tetrachloride (CCl_4_)-induced fibrotic liver models; MASH models	Reduces fibrosis	87,88
Insulin-like growth factor-1 (IGF-1) administration	Restores mitochondrial membrane potential; enhances hepatocyte proliferation	Sen-hepatocytes, sen-HSCs	CCl_4_-induced fibrotic liver models	Attenuates fibrosis	90
Alda-1/Nrf2 activation	Enhances mitochondrial function	Sen-hepatocytes, sen-HSCs	CCl_4_-induced fibrotic liver models	Attenuates fibrosis	95,98
Deferiprone/iron chelator	Reduces hepatocyte senescence	Sen-hepatocytes	CCl_4_-induced fibrotic liver models	Attenuates fibrosis	26
Senolytic agent DpC	Clears senescent cells	Sen-hepatocytes	MASLD	Reduces fibrosis	6
N-acetylcysteine (NAC)/antioxidants	Reduces reactive oxygen species (ROS) levels; inhibits senescence; promotes hepatic stellate cell (HSC) apoptosis	HSCs	CCl_4_-induced fibrotic liver models	Reduces fibrosis	96
Manganese via albumin-mediated transcytosis	Induces senescence in activated HSCs	Activated HSCs	CCl_4_-induced fibrotic liver models	Inhibits the progression of liver fibrosis	7
Programmed cell death protein 1 (PD-1)/PD-ligand 1 (PD-L1) blockade	Enhances clearance of senescent cells	Sen-hepatocytes, sen-HSCs and others	MASH models	Accelerates fibrosis regression	99,107
Simvastatin	Improves the hepatic sinusoidal microenvironment	Liver sinusoidal endothelial cells (LSECs)	Aged CCl_4_-induced fibrotic liver models	Reduction in portal hypertension and liver fibrosis	102
Lanifibranor/peroxisome proliferator-activated receptor (PPAR) agonist	Improves fenestration of LSECs	LSECs	Thioacetamide (TAA)- and common bile duct ligation (cBDL)-induced cirrhotic liver models	Reduces fibrosis	104

## 5 Summary and future perspectives

This review synthesizes the multifaceted role of cellular senescence in liver fibrosis, emphasizing that its impact is not universally pathogenic or protective but is exquisitely context-dependent, shaped by cell type, disease stage, and the molecular composition of the SASPs. While senescent hepatocytes drive disease progression through the secretion of GDF15, they concurrently recruit and activate NK cells via the CXCL-10/CXCR3 axis to facilitate immune-mediated clearance.^6^^,^^31^ Senescent HSCs reduce ECM production but propagate senescence through paracrine signaling.^14^^,^^43^ Senescent LSECs impair microcirculation.^20^^,^^51^ In addition, senescent Kupffer cells shift towards a pro-inflammatory phenotype with impaired phagocytosis, and senescent NK cells undergo functional exhaustion characterized by reduced cytotoxicity, collectively disrupting immune homeostasis.^73^ At the core of these interactions lies the SASP, which remodels the intrahepatic microenvironment. Pro-inflammatory SASP components recruit monocytes and M1 macrophages to sustain inflammation, while anti-inflammatory factors drive M2 polarization and ECM degradation.^108^ This dualistic regulation is further modulated by ECM-derived DAMPs. Collectively, these findings challenge the view of senescence as a unidirectional process, framing it instead as a tunable axis that can be targeted to tip the balance toward fibrosis resolution.

Despite these advances, translating pan-senescence-targeted therapies to the clinic faces significant hurdles. First, heterogeneity of senescent cell populations, both across etiologies and within individual patients, requires precision targeting to avoid disrupting beneficial senescence. Second, the lack of non-invasive biomarkers for senescence hinders patient stratification and monitoring of therapeutic response. Third, the long-term safety of senolytics or SASP modulators remains untested, particularly in patients with advanced cirrhosis or comorbidities. Targeting cellular senescence, whether by clearing senescent cells, modulating their SASP, or restoring their reparative functions, offers a paradigm shift in antifibrotic therapy, moving beyond HSC-centric approaches to address the full complexity of fibrotic microenvironment remodeling. Given the challenges of selective senescent cell ablation, emerging stem cell-based therapies, such as mesenchymal stem cells (MSCs), offer an avenue for restoring the diseased hepatic microenvironment.^109^ Both the cellular functions of stem cells and their paracrine mediators exert tissue-reparative effects and hold great potential for repairing aged liver tissue.^110^ Intriguingly, senescent HSCs exhibit a high degree of plasticity and contribute to tissue repair, a function extending beyond being merely a pre-apoptotic state. As part of SASPs, factors like IL-6 and CXCR2 ligands can paradoxically stimulate liver regeneration by activating signaling pathways such as Hippo-YAP.^111^ This underscores that cellular senescence is not simply a terminal fate but a dynamic cell state capable of orchestrating complex tissue responses. However, in the context of fibrosis or cirrhosis, the indiscriminate activation of regenerative pathways, driven by persistent SASP factors such as IL-6, may paradoxically fuel aberrant hepatocyte proliferation, disrupt tissue architecture, and ultimately precipitate hepatocellular carcinoma (HCC).^112^^,^^113^ Consequently, future therapeutic strategies may evolve beyond the mere ablation of senescent cells to instead harness their pro-regenerative potential, thereby promoting repair following liver injury.

## Authors' contributions

**Yuqi Gu:** Writing – original draft, Writing – review & editing. **Linjing Zhan:** Writing – original draft，Writing – review & editing. **Yaping Di:** Writing – original draft. **Haipeng Wu:** Writing – review & editing. **Tiejun Zhao:** Supervision, Funding acquisition, Project administration. **Fei Zhang:** Conceptualization, Writing – review & editing, Supervision, Funding acquisition, Project administration. **Qirou Wu:** Conceptualization, Writing – original draft, Writing – review & editing, Supervision, Funding acquisition, Project administration.

## AI declaration

Qwen (Tongyi Qianwen, Alibaba Group) was used solely to check the English grammar and correct typographical errors throughout the manuscript. The authors are responsible for the integrity of the whole research.

## Declaration of competing interest

The authors declare that there is no conflicts of interest.
